# Nicotine metabolite ratio and clinically significant depressive symptoms in U.S. adults: A smoker-specific association

**DOI:** 10.1097/MD.0000000000048186

**Published:** 2026-04-10

**Authors:** Ying Zhu, Ruibo Miao

**Affiliations:** a Department of Neurosurgery, The First Hospital of China Medical University, Shenyang, China.

**Keywords:** depression, multiple imputation, nicotine metabolite ratio, PHQ-9, smokers, survey-weighted

## Abstract

Nicotine metabolite ratio (NMR) is a biomarker for the rate of nicotine metabolism and may be linked to depression, but evidence from large, representative populations is scarce. The role of smoking status as a potential effect modifier of this association is poorly understood. Identifying smoker-specific vulnerability may inform risk stratification and integration of mental-health screening within smoking-cessation settings. We aimed to investigate the association between NMR and clinically significant depressive symptoms in U.S. adults and among active smokers. We conducted a cross-sectional, observational analysis of 9287 adults (≥20 years) from the National Health and Nutrition Examination Survey 2013 to 2018. The exposure was the natural ln(NMR). The outcome was clinically significant depressive symptoms (Patient Health Questionnaire-9 [PHQ-9] score ≥10). We used survey-weighted modified Poisson regression to estimate relative risks with 95% confidence intervals; missing covariates were addressed using multiple imputation. Analyses were stratified by smoking status using a union definition (self-report or serum cotinine ≥3 ng/mL); robustness was evaluated using a stricter threshold of ≥10 ng/mL. In the overall population, ln(NMR) was not associated with depressive symptoms (per 1- standard deviation [SD] increase, RR 1.03; 95% CI 0.93–1.14). However, among smokers (union definition; cotinine ≥3 ng/mL), a higher ln(NMR) was associated with a higher prevalence of clinically significant depressive symptoms (RR per 1-SD, 1.15; 95% CI, 1.02–1.29). This finding was robust using a stricter cotinine cutoff of ≥10 ng/mL (RR per 1-SD, 1.16; 95% CI, 1.02–1.32). Restricted cubic splines revealed a near-linear dose–response relationship in smokers only. Significant interactions were observed across multiple covariates – including body mass index, poverty-income ratio, race/ethnicity, sex, smoking status, hypertension, diabetes, cotinine level, and stroke – after false discovery rate control. In this study, higher ln(NMR) was associated with a higher prevalence of clinically significant depressive symptoms among active smokers, but not in the overall U.S. adult. These findings highlight a smoker-specific vulnerability and suggest that NMR may be useful for risk stratification to support integrated smoking-cessation and mental-health screening; longitudinal and interventional studies are needed to clarify directionality and clinical utility.

**Highlights**

- In 9287 U.S. adults from NHANES 2013 to 2018, ln(NMR) showed no association with depressive symptoms overall.
- Among active smokers (union; cotinine ≥3 ng/mL), higher ln(NMR) was associated with higher risk of PHQ-9 ≥10 (per-SD RR ≈ 1.15); results held at ≥10 ng/mL.
- Dose–response was near-linear in smokers and absent overall (*P* for nonlinearity >0.29 in smokers).
- Effect modification among smokers was observed for BMI, PIR, race/ethnicity, sex, smoking status, hypertension, diabetes, baseline cotinine level, and stroke (BH-FDR ≤0.002); no interaction for age, alcohol use, or CHD).
- Findings suggest that links between nicotine metabolism and depression are contingent on active nicotine exposure, informing risk stratification for cessation and mental-health screening.

## 1. Introduction

Tobacco smoking and depression frequently co-occur and constitute a major public health concern.^[1]^ People with clinically diagnosed depressive disorders have 2 to 3 times the smoking prevalence of the general population, face greater difficulty quitting, and bear excess tobacco-related morbidity and mortality.^[2,3]^ The link is bidirectional and multifactorial, reflecting shared genetic liability, self-medication of mood symptoms, and neurobiological adaptations.^[4]^ Nicotine – the principal psychoactive constituent of tobacco – acutely modulates dopaminergic and serotonergic systems implicated in mood regulation and depressive psychopathology.^[5,6]^ Importantly, depressive symptoms captured by screening instruments are conceptually distinct from clinically diagnosed depressive disorders; symptom burden may fluctuate with recent exposures and withdrawal, whereas diagnoses such as major depressive disorder (MDD) require structured clinical assessment.

Nicotine metabolism varies widely between individuals.^[7,8]^ Hepatic CYP2A6 converts nicotine → cotinine → 3-hydroxycotinine (3HC); the nicotine metabolite ratio (NMR; 3HC/cotinine) is a validated, relatively stable in vivo proxy of CYP2A6 activity and tracks total nicotine clearance.^[9,10]^ NMR is shaped by genetic variation (CYP2A6 polymorphisms) and by demographic/physiologic factors such as sex, race/ethnicity, and body mass index (BMI).^[11,12]^

Compared with slow metabolizers, fast metabolizers (higher NMR) clear nicotine more rapidly, show greater dependence and compensatory smoking to avert withdrawal, and experience steeper between-cigarette declines in nicotine.^[13]^ Withdrawal features – irritability, anxiety, poor concentration, and depressed mood – overlap with depressive symptom domains assessed by the Patient Health Questionnaire-9 (PHQ-9), which quantifies symptom frequency over the prior 2 weeks and is commonly used to identify clinically significant depressive symptom burden (e.g., PHQ-9 ≥10) rather than to establish a formal psychiatric diagnosis.^[14]^ Thus, we hypothesize that higher NMR may be associated with greater depressive symptom burden specifically among active smokers, consistent with a withdrawal-related vulnerability framework.^[15]^

Despite this compelling biological rationale, epidemiological evidence remains inconsistent. Some clinical studies report no mean NMR difference between smokers with and without major depressive disorder.^[16]^ This may reflect outcome heterogeneity (MDD diagnosis vs symptom severity) and setting differences. Mendelian-randomization (MR) analyses implicate smoking heaviness in depression risk, potentially independent of circulating nicotine and involving non-nicotine smoke constituents^[17]^; yet such designs capture lifelong genetic liability, not the short-term withdrawal dynamics most relevant to active smokers.^[18]^ Population-based evidence that jointly leverages objective NMR, standardized symptom screening (e.g., PHQ-9), and formal tests of smoking-status effect modification remains limited, leaving uncertainty about whether any NMR–depression link is smoker-specific or generalizable to the broader adult population.

To address these gaps, this study leverages data from a large, nationally representative sample of U.S. adults to investigate the association between NMR and clinically significant depressive symptoms. The primary objective is to test whether smoking status fundamentally modifies this association, positing that the relationship is present among active smokers but null in the overall population. Relative to prior clinical studies focused on selected treatment-seeking samples and MDD diagnoses, and genetic studies capturing lifelong liability, our approach provides biomarker-based, survey-weighted population evidence and directly evaluates smoker-specific associations and dose–response patterns using biochemically anchored smoking definitions.

## 2. Methods

### 2.1. Study design and data source

We conducted a cross-sectional analysis of 3 consecutive National Health and Nutrition Examination Survey (NHANES) cycles (2013–2014, 2015–2016, 2017–2018). NHANES uses a complex, multistage probability design to sample the civilian, noninstitutionalized U.S. population and combines household interviews with standardized examinations in mobile examination centers. The NHANES protocol was approved by the National Center for Health Statistics Research Ethics Review Board; all participants provided written informed consent. The present study analyzed de-identified public data and required no additional institutional review.

### 2.2. Study population

**Figure 1.**
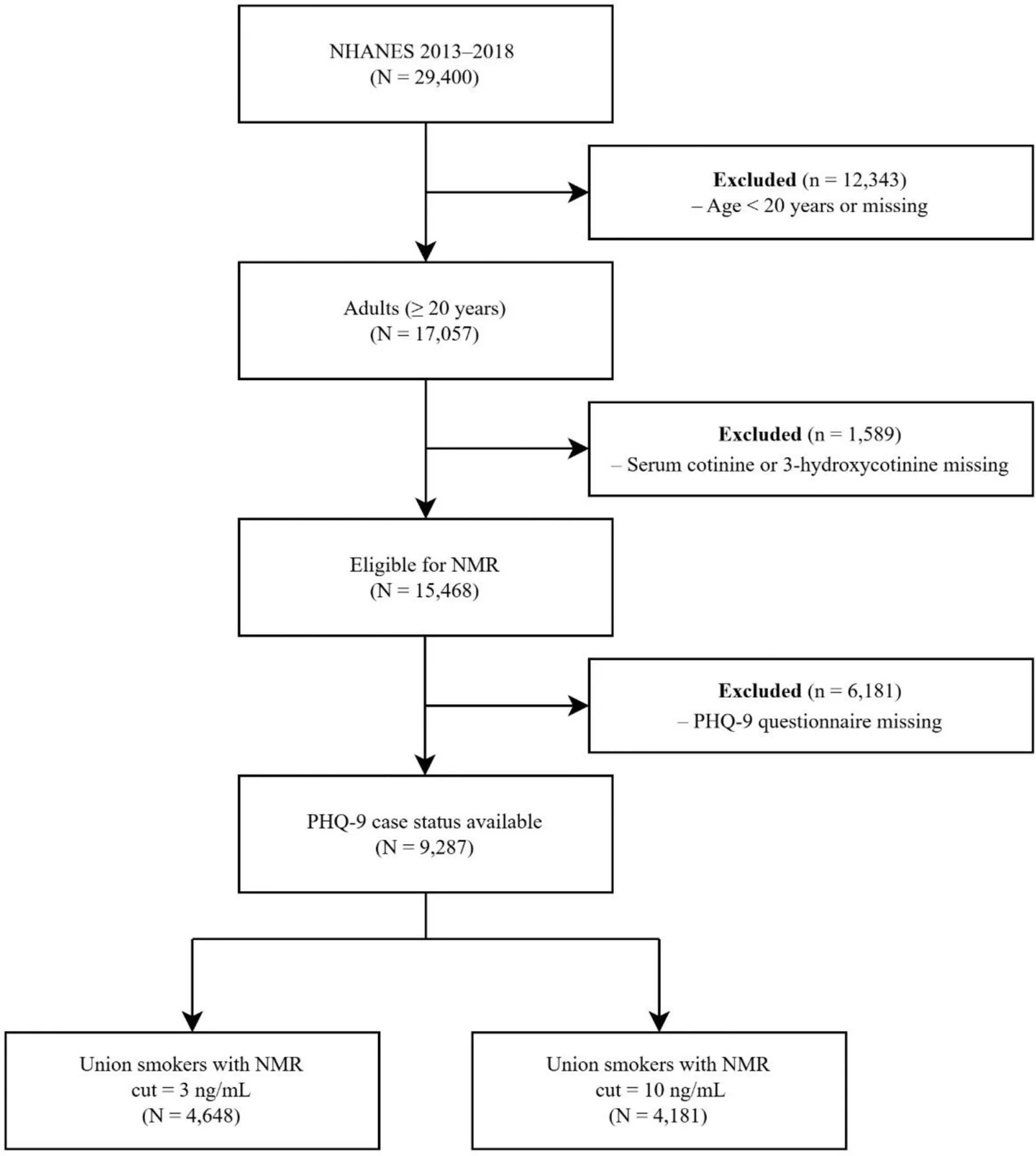
Flow diagram of participant selection. Participant flow diagram (NHANES 2013–2018) showing exclusions and final analytic samples for the overall population and for smoker subgroups (union rule; cotinine cutoffs 3 and 10 ng/mL).NHANES = National Health and Nutrition Examination Survey.

Across the 3 cycles, 29,400 participants were examined. As detailed in Figure 1, we excluded individuals <20 years or with missing age (n = 12,343), leaving 17,057 adults. We then excluded participants missing either serum cotinine or 3HC required for computing NMR (n = 1589) and those missing PHQ-9 (n = 6181). The final analytic sample comprised 9287 adults for the overall analysis. Among the 9287 adults, 4648 were classified as smokers under the union definition (self-report or cotinine ≥3 ng/mL), and 4181 under a stricter definition (self-report or cotinine ≥10 ng/mL).

### 2.3. Exposure: nicotine metabolite ratio

Serum cotinine and 3HC were quantified at the CDC Division of Laboratory Sciences using solid-phase extraction and high-performance liquid chromatography–tandem mass spectrometry with isotope-dilution calibration under rigorous internal and external quality control. NMR was calculated as the molar ratio 3HC/cotinine. Because NMR is right-skewed, we used its natural logarithm [ln(NMR)] in all models to improve normality and stabilize variance.

### 2.4. Outcome: depressive symptoms

Depressive symptoms were assessed with the PHQ-9, which scores the frequency of 9 DSM-IV depression symptoms over the prior 2 weeks (0–27). The primary outcome was clinically significant depressive symptoms, defined as PHQ-9 ≥10 (binary).

### 2.5. Smoking status (effect-modifier definition)

To evaluate effect modification by active smoking, we classified smokers using a sensitive “union” definition: self-reported current smoking or serum cotinine above a prespecified threshold. The primary analysis used a union definition (self-report or serum cotinine ≥3 ng/mL); a stricter threshold of ≥10 ng/mL was used in sensitivity analyses. All nonsmokers failed both criteria.

### 2.6. Covariate assessment

Covariates were selected a priori based on prior literature and directed acyclic graph considerations, focusing on factors plausibly associated with both nicotine metabolism (ln[NMR]) and depressive symptoms, or with smoking behaviors that could confound the association. Sociodemographic variables included age (years), sex, race/ethnicity (Mexican American, Other Hispanic, Non-Hispanic White, Non-Hispanic Black, Other/Multiracial), education (<9th grade, 9–11th grade, high school/GED, some college/AA, college graduate+), poverty-income ratio (PIR; continuous), and survey cycle (2013–2014, 2015–2016, 2017–2018). Lifestyle/behavioral variables included self-reported smoking status (never/former/current) and alcohol use in the past 12 months (yes/no). Self-reported smoking status was retained to capture longer-term behavioral context beyond point-in-time biochemical exposure. To disentangle metabolic rate from exposure intensity, ln(cotinine) was included as an adjustment covariate to account for recent nicotine intake; this strategy helps reduce residual confounding by smoking dose so that the ln(NMR) association can be interpreted conditional on a similar level of recent exposure. Clinical variables included BMI (kg/m^2^), diabetes, hypertension, coronary heart disease (CHD), and stroke (physician-diagnosed; all binary). When cotinine was used as a stratifier (e.g., tertiles), ln(cotinine) was omitted from the adjustment to avoid collinearity.

### 2.7. Statistical analysis

Analyses were performed in R 4.5.1. The NHANES complex design was incorporated using Taylor-series linearization with strata, primary sampling units, and examination weights (WTMEC2YR divided by 3 for 2013–2018) via the survey package; singleton PSUs were handled with the package default adjustment. Missing covariate data were addressed with multiple imputation by chained equations (mice, 10 imputations). Imputation models included the exposure, outcome, design variables, and all analysis covariates; estimates were pooled using Rubin rules (via mitools:MIcombine) to obtain combined point estimates and standard errors.

We fitted survey-weighted modified Poisson models with a log link and robust (sandwich) variance to estimate relative risks and 95% confidence intervals for ln(NMR) in relation to PHQ-9 ≥10. The fully adjusted model included sociodemographic factors (age, sex, race/ethnicity, education, PIR, survey cycle), lifestyle/behavioral factors (self-reported smoking status, alcohol use), clinical factors (BMI, diabetes, hypertension, CHD, stroke), and ln(cotinine) to account for recent exposure intensity. The exposure was parameterized 3 ways – quartiles (Q1 reference) with a linear trend across quartiles using their within-quartile medians, and a standardized continuous specification (per 1- standard deviation of ln[NMR]). For comparability, quartile cut-points and the standard deviation of ln(NMR) were computed within each analytic population and within each imputed dataset, with estimates pooled across imputations.

Dose–response and nonlinearity were evaluated using restricted cubic splines with 4 knots at the 5th, 35th, 65th, and 95th percentiles of ln(NMR), using the median as the reference; overall association and departures from linearity were tested with Wald tests of spline terms. Effect modification was prespecified for smoking status, with additional subgroup analyses by age, sex, race/ethnicity, BMI, PIR, alcohol use, hypertension, diabetes, CHD, stroke and cotinine level (tertiles). Multiplicative interactions were assessed by adding product terms between ln(NMR) and the stratifier in fully adjusted models; false discovery rate was controlled using the Benjamini–Hochberg (BH) procedure for interaction *P*-values. Two-sided α = 0.05 denoted statistical significance. Results are presented with forest plots (subgroups) and restricted cubic splines curves (dose–response).

## 3. Results

### 3.1. Participant selection and sample characteristics

Across the 2013 to 2018 survey cycles, 29,400 participants were examined. After excluding individuals aged <20 years or with missing age (n = 12,343), 17,057 adults remained. We further excluded those missing either serum cotinine or 3HC (n = 1589) and those without PHQ-9 data (n = 6181), yielding a final analytic sample of 9287 adults for the overall analysis. Two smoker subgroups were defined from this sample using the union definition: 4648 participants at cotinine ≥3 ng/mL and 4181 at cotinine ≥10 ng/mL (Fig. 1).

In the primary smoker subgroup (cotinine ≥ 3 ng/mL), higher NMR quartiles marked a distinct profile (Table 1): participants were older (about + 8 years, 37.5→45.9), more often female (34%→52%) and Non-Hispanic White (48%→77%), with slightly lower BMI (~29→28 kg/m^2^) and more hypertension (28%→40%). Baseline depressive symptoms did not differ meaningfully across quartiles (PHQ-9 mean ≈ 4.1–4.6; severity distribution *P* = .487), suggesting that crude symptom burden alone does not explain subsequent associations. When stratified by depression status (Table S1, Supplemental Digital Content, https://links.lww.com/MD/R620), smokers with PHQ-9 ≥10 showed a slightly higher ln(NMR) (−0.88 vs − 0.98; *P *= .016), were more often female (59% vs 37%) and current smokers (82% vs 66%), and had a lower PIR (1.90 vs 2.52). Together these features are consistent with a more vulnerable clinical and socioeconomic profile among those with clinically significant symptoms. These cross-sectional patterns were replicated under a stricter smoker definition (cotinine ≥10 ng/mL; Table S2, Supplemental Digital Content, https://links.lww.com/MD/R620): from the lowest to highest NMR quartile, age increased by approximately 7 years and the female proportion by about 20 percentage points, while PHQ-9 means again showed no clear gradient (≈4.1–4.7). For broader context in the overall adult population (Table S3, Supplemental Digital Content, https://links.lww.com/MD/R620), higher NMR quartiles corresponded to older age (≈34→55 years) and a larger Non-Hispanic White share (≈48%→82%), with PHQ-9 totals lowest in the mid-range quartile, reinforcing that the primary gradients across NMR reflect demographic and cardiometabolic composition rather than baseline symptom load.

**Table 1:** Baseline characteristics by NMR quartiles among smokers (union; cut = 3 ng/mL).

Characteristic	Overall, N = 4,648*	Q1 (lowest), N = 1162*	Q2, N = 1162*	Q3, N = 1162*	Q4 (highest), N = 1162*	*P*
NMR (3HC/cotinine)	0.45 (0.29)	0.16 (0.05)	0.30 (0.03)	0.43 (0.04)	0.79 (0.31)	<.001
ln(NMR)	−0.98 (0.64)	−1.90 (0.49)	−1.22 (0.12)	−0.85 (0.10)	−0.29 (0.30)	<.001
PHQ-9 total score	4.32 (5.05)	4.37 (5.08)	4.06 (4.81)	4.23 (4.86)	4.56 (5.38)	.564
PHQ-9 severity						
None (0–4)	1659 (65%)	398 (66%)	411 (66%)	423 (65%)	427 (64%)	.487
Mild (5–9)	528 (21%)	132 (22%)	130 (22%)	135 (20%)	131 (19%)	
Moderate (10–14)	203 (8.8%)	38 (6.3%)	48 (7.0%)	52 (9.9%)	65 (11%)	
Moderately severe (15–19)	99 (3.9%)	22 (3.5%)	23 (4.1%)	19 (2.7%)	35 (5.1%)	
Severe (20–27)	49 (1.8%)	13 (2.4%)	10 (1.1%)	12 (1.7%)	14 (1.9%)	
Age (year)	41.47 (16.54)	37.51 (16.26)	39.21 (16.32)	41.64 (16.01)	45.85 (16.35)	<.001
Sex						
Male	2786 (59%)	767 (66%)	741 (64%)	703 (60%)	575 (48%)	<.001
Female	1862 (41%)	395 (34%)	421 (36%)	459 (40%)	587 (52%)	
Race/ethnicity						
Mexican American	451 (6.7%)	103 (7.9%)	113 (7.2%)	124 (6.8%)	111 (5.2%)	<.001
Other Hispanic	320 (4.6%)	67 (4.7%)	84 (5.5%)	84 (4.7%)	85 (3.7%)	
Non-Hispanic White	1948 (65%)	304 (48%)	456 (61%)	534 (68%)	654 (77%)	
Non-Hispanic Black	1394 (16%)	484 (27%)	404 (20%)	297 (12%)	209 (8.1%)	
Other/multiracial	535 (8.2%)	204 (12%)	105 (6.8%)	123 (8.4%)	103 (6.2%)	
Education						
<9th grade	282 (4.0%)	63 (5.0%)	66 (4.0%)	68 (3.2%)	85 (4.1%)	.047
9–11th grade	763 (15%)	196 (18%)	184 (16%)	205 (14%)	178 (12%)	
High school/GED	1226 (33%)	292 (30%)	296 (33%)	320 (36%)	318 (31%)	
Some college/AA	1372 (35%)	306 (33%)	336 (34%)	357 (34%)	373 (37%)	
College graduate+	460 (14%)	115 (14%)	97 (13%)	101 (13%)	147 (17%)	
PIR	2.35 (1.59)	2.13 (1.51)	2.27 (1.58)	2.40 (1.59)	2.51 (1.64)	<.001
Marital status						
Married	1513 (40%)	345 (39%)	344 (39%)	390 (39%)	434 (43%)	.002
Living with partner	553 (14%)	134 (13%)	140 (15%)	149 (13%)	130 (13%)	
Never married	1047 (25%)	319 (32%)	280 (27%)	239 (24%)	209 (20%)	
Widowed	213 (4.1%)	27 (2.1%)	31 (2.3%)	62 (5.2%)	93 (5.6%)	
Divorced	585 (13%)	110 (10%)	132 (12%)	163 (15%)	180 (15%)	
Separated	194 (3.9%)	38 (3.4%)	52 (3.9%)	49 (4.0%)	55 (4.2%)	
Any alcohol use in past 12 mo	3278 (86%)	788 (87%)	782 (86%)	852 (86%)	856 (85%)	.687
Smoking status						
Never†	688 (14%)	203 (17%)	202 (17%)	151 (14%)	132 (11%)	.020
Former	594 (16%)	135 (17%)	127 (13%)	144 (15%)	188 (20%)	
Current	3017 (69%)	694 (66%)	716 (70%)	795 (70%)	812 (70%)	
BMI (kg/m^2^)	28.71 (7.35)	28.91 (7.64)	29.35 (7.90)	28.63 (7.20)	28.13 (6.75)	.004
Waist circumference (cm)	98.41 (18.19)	97.79 (18.61)	99.69 (19.98)	98.76 (18.13)	97.49 (16.26)	.239
Sleep duration (hours/night)	7.28 (1.68)	7.21 (1.69)	7.18 (1.75)	7.22 (1.63)	7.45 (1.67)	.008
Hypertension (ever told)	1471 (32%)	309 (28%)	303 (28%)	362 (29%)	497 (40%)	<.001
Diabetes (doctor told)						
No	4083 (90%)	1049 (92%)	1055 (92%)	1013 (91%)	966 (86%)	.002
Yes	467 (8.3%)	92 (6.7%)	89 (7.0%)	120 (6.7%)	166 (12%)	
Borderline	96 (1.8%)	21 (1.8%)	17 (1.1%)	29 (2.2%)	29 (2.0%)	
History of stroke	184 (3.3%)	51 (3.9%)	24 (2.1%)	39 (2.5%)	70 (4.5%)	.044
History of CHD	156 (3.7%)	25 (2.5%)	26 (2.5%)	36 (3.9%)	69 (5.3%)	.040

3HC = 3-hydroxycotinine, BMI = body mass index, CHD = coronary heart disease, NMR = nicotine metabolite ratio, PHQ-9 = Patient Health Questionnaire-9, PIR = poverty-income ratio.

* Values are survey-weighted mean (SE) for continuous variables or survey-weighted % for categorical variables; unweighted n are shown. *P* values were obtained from survey-weighted linear regression (continuous) or Rao–Scott χ^2^ tests (categorical).

† Self-reported smoking status is shown for behavioral context; the smoker subpopulation is defined by the union rule (self-report or cotinine ≥3 ng/mL), so “never/former” can appear within this subpopulation.

### 3.2. Main association

In the overall adult population, ln(NMR) was not associated with clinically significant depressive symptoms (per-SD RR 1.03, 95% CI 0.93–1.14), and the quartile analysis showed no trend (*P* for trend = .401). In contrast, among smokers, higher ln(NMR) related to higher risk. For the primary definition (cotinine ≥3 ng/mL), the per-SD RR was 1.15 (95% CI 1.02–1.29) with a significant per-quartile trend (*P* = .031). Findings were replicated using the stricter definition (cotinine ≥10 ng/mL: per-SD RR 1.16, 95% CI 1.02–1.32; per-quartile trend *P* = .030). Q4 vs Q1 comparisons were directionally consistent and borderline significant in both smoker groups. Full estimates are reported in Table 2.

**Table 2:** Association between ln(NMR) and PHQ-9 ≥10 in overall adults and smokers.

Contrast	Overall		Smokers (cut = 3)		Smokers (cut = 10)	
	RR (95% CI)	*P*	RR (95% CI)	*P*	RR (95% CI)	*P*
Q1 (ref)	Reference	–	Reference	–	Reference	–
Q2 versus Q1	1.02 (0.79–1.32)	.881	1.00 (0.68–1.47)	.988	0.95 (0.64–1.40)	.778
Q3 versus Q1	1.05 (0.81–1.34)	.724	1.22 (0.80–1.85)	.352	1.13 (0.72–1.76)	.607
Q4 versus Q1	1.29 (0.83–2.01)	.249	1.31 (0.98–1.73)	.065	1.32 (0.99–1.77)	.063
Per-quartile increase	1.05 (0.94–1.17)	.401	1.11 (1.01–1.21)	.031	1.12 (1.01–1.23)	.030
ln(NMR), per 1-SD	1.03 (0.93–1.14)	.598	1.15 (1.02–1.29)	.021	1.16 (1.02–1.32)	.023

*P* for trend: overall = 0.401; cut = 3, 0.031; cut = 10, 0.030.

Quartile models use Q1 as the reference category. “per quartile increase” represents the linear trend across quartiles (Q1–Q4).

CI = confidence interval, NMR = nicotine metabolite ratio, PHQ-9 = Patient Health Questionnaire-9, RR = relative risk.

### 3.3. Dose–response

**Figure 2.**
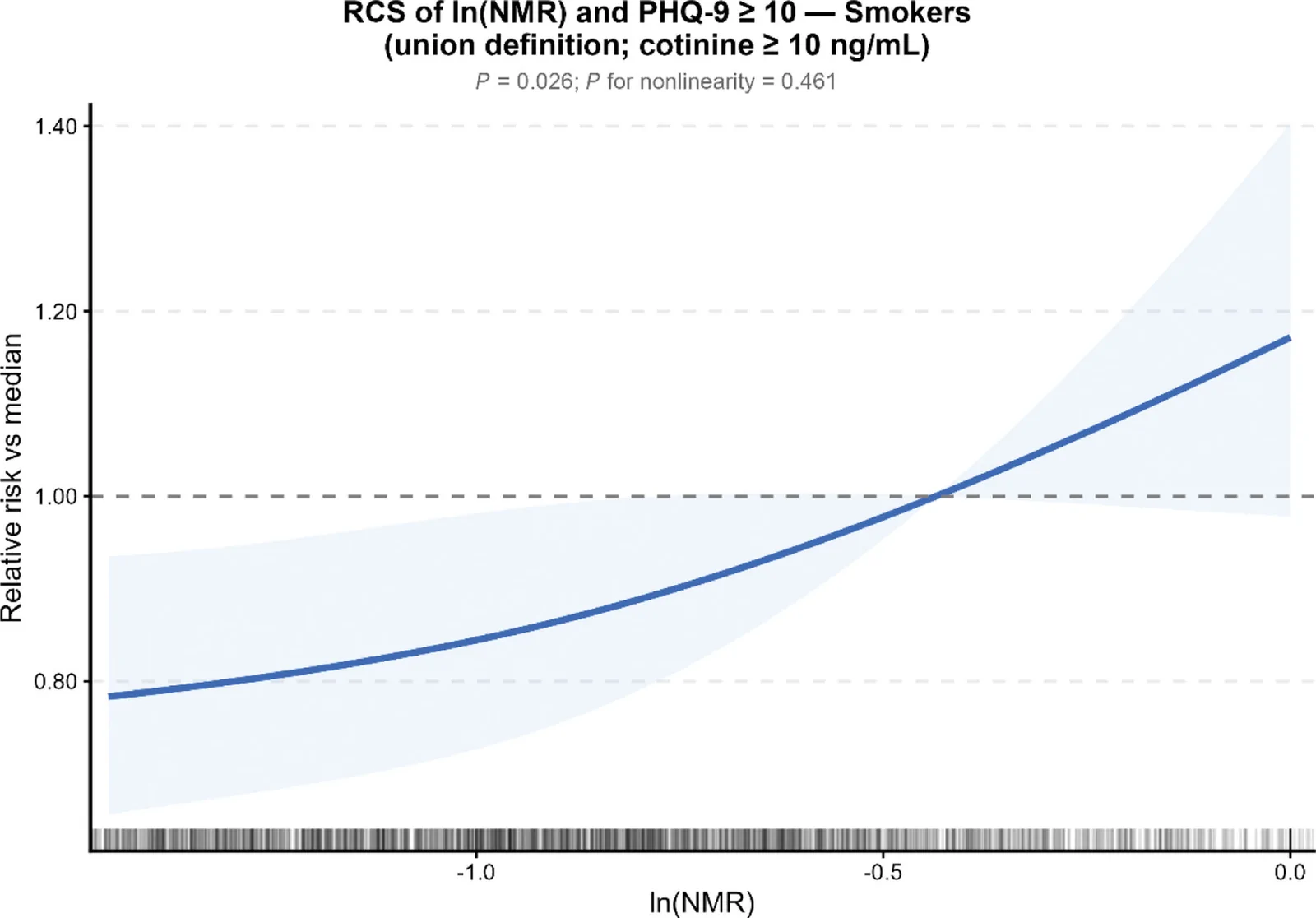
Restricted cubic spline of ln(NMR) versus PHQ-9 ≥10 among smokers (union; cotinine ≥3 ng/mL); risk shown relative to the median under a fully adjusted, survey-weighted model. NMR = nicotine metabolite ratio, PHQ = Patient Health Questionnaire.

In smokers, restricted cubic spline curves rose monotonically across ln(NMR) with no evidence of curvature. Under the primary definition (Fig. 2; cotinine ≥3 ng/mL), the association was significant (overall *P* = .026) and approximately linear (*P* for nonlinearity = 0.461). The stricter definition reproduced the same shape (Fig. S1, Supplemental Digital Content, https://links.lww.com/MD/R621; cotinine ≥10 ng/mL: overall *P* = .023; *P* for nonlinearity = .295).

In the overall adult sample, the curve was essentially flat around unity across the observed range (Fig. S2, Supplemental Digital Content, https://links.lww.com/MD/R621), and both tests were null (overall *P* = .659; *P* for nonlinearity = .492). Taken together, the spline evidence indicates that the dose–response is confined to active smokers and is well approximated by a linear trend, consistent with the per-SD estimates in Table 2.

### 3.4. Subgroup and interaction analyses

**Figure 3.**
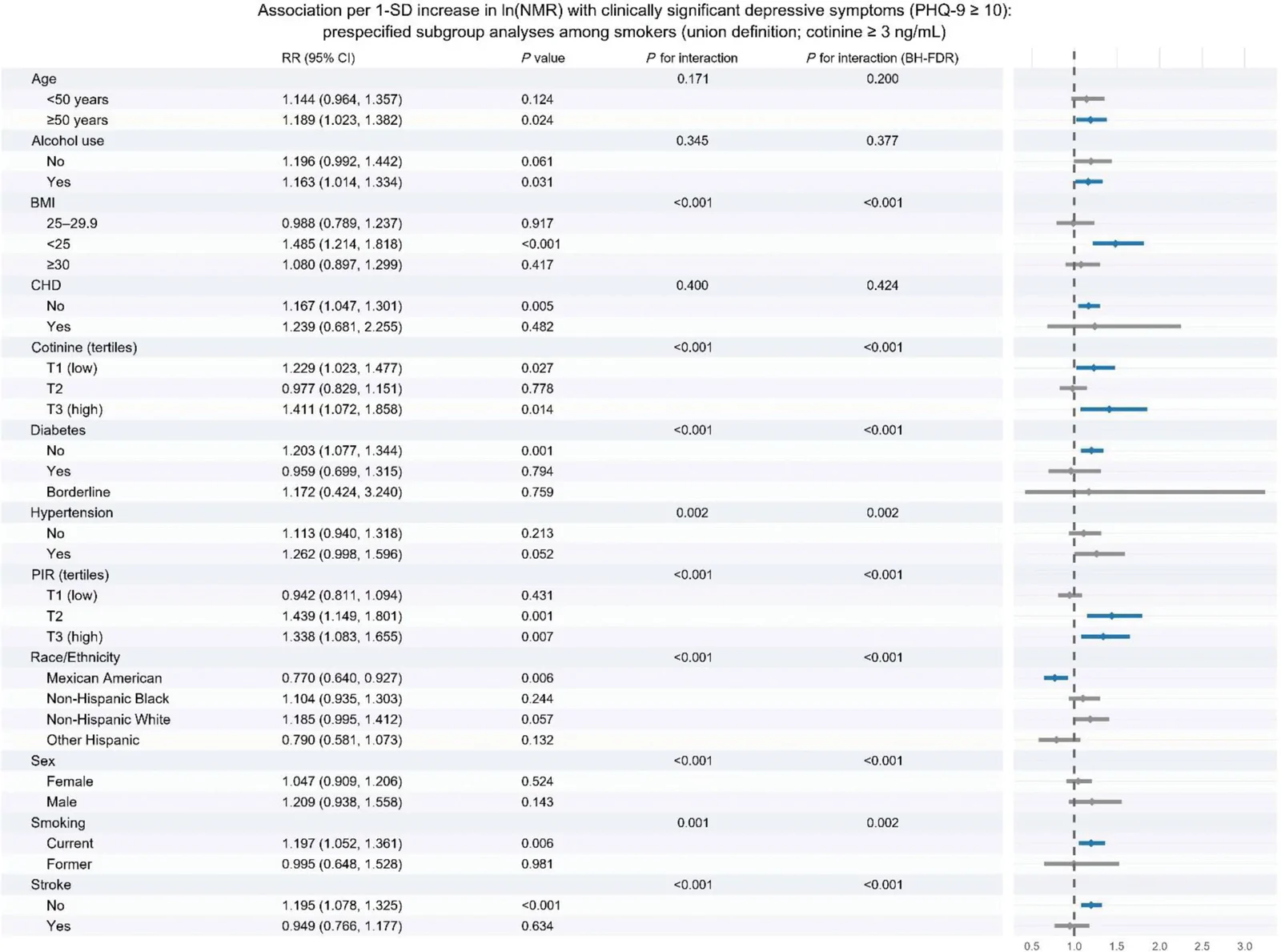
Prespecified subgroup analyses among smokers (union definition; serum cotinine ≥3 ng/mL): forest plot of per-SD ln(NMR) relative risks with *P* for interaction (raw and BH-FDR). BH = Benjamini–Hochberg, FDR = false discovery rate, NMR = nicotine metabolite ratio, SD = standard deviation.

Figure 3 (smokers, cotinine ≥3 ng/mL) is the primary subgroup analysis. The forest plot shows stratum-specific relative risks per 1-SD higher ln(NMR) (with 95% confidence intervals) from fully adjusted models. *P* for interaction tests multiplicative effect modification across strata; BH-FDR-adjusted values are provided to account for multiple comparisons. The positive association of ln(NMR) with PHQ-9 ≥10 persisted across most strata but varied in magnitude, indicating effect modification. Interactions were observed for BMI, PIR, race/ethnicity, sex, smoking status, and hypertension (*P* for interaction and BH-FDR-adjusted values shown in Fig. 3), indicating that the ln(NMR)–PHQ-9 ≥10 association differed in magnitude across these strata. Effects were strongest in lean participants (BMI <25 kg/m^2^) and in those with higher PIR, and were more apparent among current than former smokers. Several strata also showed illustrative point estimates – for example, among smokers without diabetes, the per-SD RR exceeded 1.20, whereas estimates were attenuated in those with diabetes – consistent with a smoker-specific gradient amplified by physiologic and socioeconomic context.

These patterns were reproduced when smoking was defined more strictly (cotinine ≥ 10 ng/mL; Fig. S3, Supplemental Digital Content, https://links.lww.com/MD/R621), with similar directions and magnitudes of interaction. By comparison, the overall adult population (Fig. S4, Supplemental Digital Content, https://links.lww.com/MD/R621) showed no coherent subgroup pattern in the absence of a main effect, reinforcing that heterogeneity signals in Figure 3 reflect modification of a smoker-specific association rather than spurious subgroup differences.

### 3.5. Sensitivity analyses

**Figure 4.**
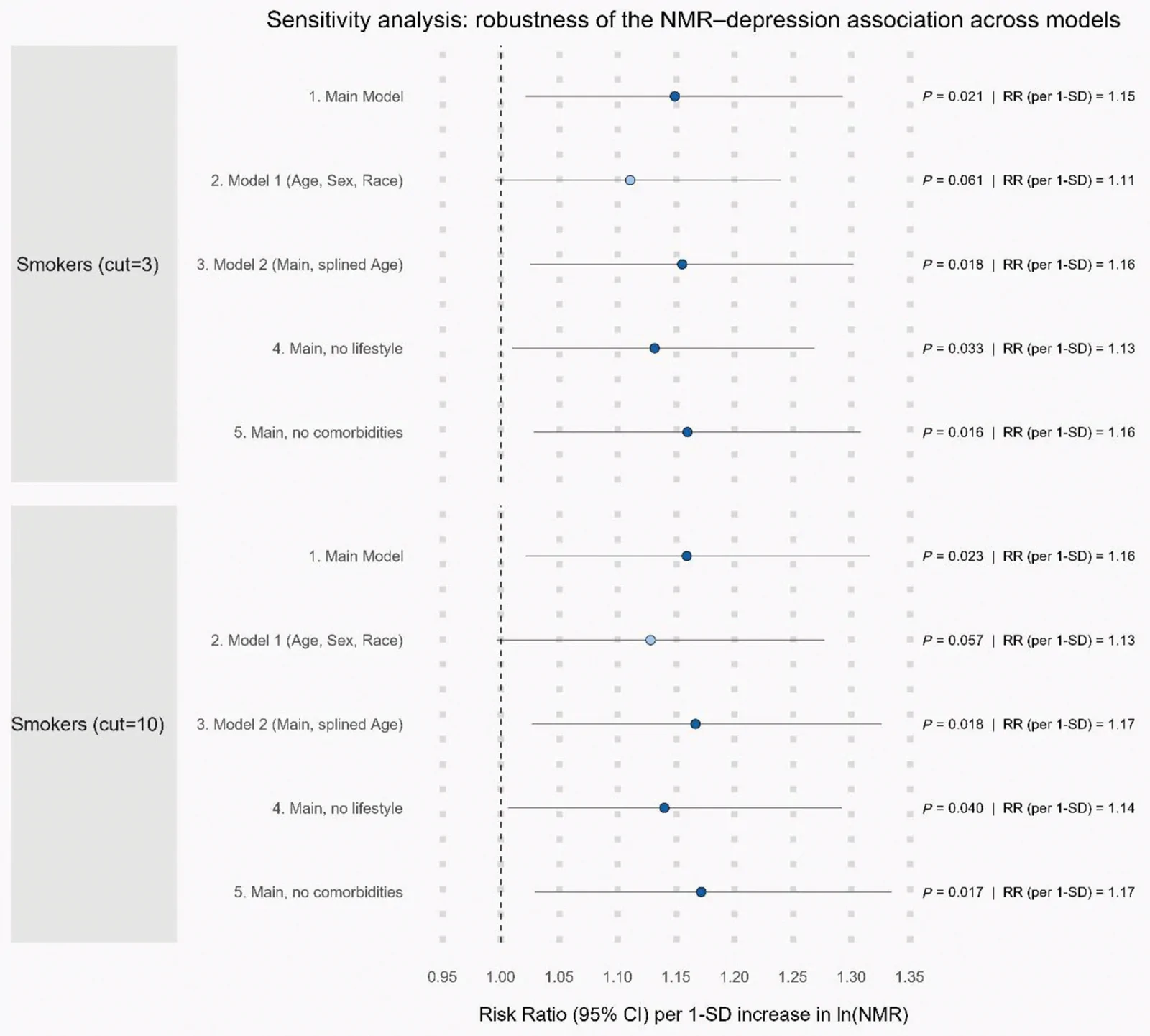
Sensitivity analyses across alternative model specifications: forest plot of per-SD ln(NMR) estimates shown separately for smokers (cut = 3 ng/mL) and smokers (cut = 10 ng/mL). NMR = nicotine metabolite ratio, SD = standard deviation.

Results were robust to alternative covariate specifications and functional forms (Fig. 4). Among smokers with cotinine ≥3 ng/mL, the per-SD association of ln(NMR) with PHQ-9 ≥10 in the main model (RR = 1.15; *P* = .021) changed little when age was modeled flexibly (RR = 1.16; *P* = .018), lifestyle variables were removed (RR = 1.13; *P* = .033), or comorbidities were removed (RR = 1.16; *P* = .016). As expected, a minimally adjusted model (age/sex/race only) attenuated the estimate but preserved direction (RR = 1.11; *P* = .061).

Using the stricter smoker definition (cotinine ≥10 ng/mL) yielded an almost identical pattern: main model RR = 1.16 (*P* = .023); splined age RR = 1.17 (*P* = .018); no lifestyle RR = 1.14 (*P* = .040); no comorbidities RR = 1.17 (*P* = .017); and the minimally adjusted model RR = 1.13 (*P* = .057).

Together, these checks indicate that the observed smoker-specific association is not driven by modeling choices or any single block of covariates; if anything, omission of lifestyle or comorbidity adjustments slightly strengthened the association, and reduced adjustment mainly attenuated precision rather than reversing the effect.

## 4. Discussion

### 4.1. Principal findings

This study, based on a large, nationally representative sample of U.S. adults, showed clear effect modification by smoking status in the relationship between the rate of nicotine metabolism and depressive symptoms. The principal finding is twofold: first, in the overall adult population, there is no association between NMR and the prevalence of clinically significant depressive symptoms. Second, among active smokers, a faster rate of nicotine metabolism, as indicated by a higher NMR, is robustly and linearly associated with an increased risk of such symptoms.

### 4.2. Biological plausibility and interpretation of smoker-specific effects

The stark dichotomy in findings between the general population and active smokers suggests that the observed association is contingent upon ongoing, cyclical nicotine exposure; however, mechanistic interpretations remain hypothesis-generating in this cross-sectional analysis. The null result in the overall population, which is predominantly composed of nonsmokers and former smokers, suggests that NMR is not an intrinsic, standalone biomarker for depression risk. If the CYP2A6 gene had pleiotropic effects on brain chemistry predisposing to depression, one might expect to see at least an attenuated signal in the full population. Its absence is less consistent with a direct, substrate-independent link, although alternative explanations (e.g., measurement limitations or residual confounding) cannot be excluded.^[19]^

Instead, the pattern is consistent with an interpretation in which any relevance of NMR to mood may depend on active smoking behavior and exposure dynamics. Our models adjusted for ln(cotinine), separating metabolic rate from recent exposure intensity. Biologically, faster metabolizers experience steeper between-cigarette declines in nicotine, precipitating more frequent/earlier withdrawal.^[20]^ One plausible explanation is a nicotine withdrawal-related framework.^[21]^ Fast metabolizers (high NMR) clear nicotine more rapidly, leading to a decline in plasma nicotine levels below the threshold required to prevent withdrawal symptoms more quickly and frequently between cigarettes.^[22]^ This model provides a coherent interpretation for why the association is confined to individuals who are actively maintaining a state of nicotine dependence. Notably, withdrawal severity, dependence symptoms, and quit attempts were not directly measured here; thus, mechanistic pathways cannot be confirmed and should be tested in longitudinal or experimental studies.

### 4.3. Synthesis with existing epidemiological and clinical research

Our findings help to reconcile seemingly contradictory results in the existing literature. Previous studies based on clinical trial populations reported no significant difference in NMR between smokers with a formal diagnosis of MDD and nondepressed smokers.^[14,23]^ The present study does not contradict those findings but rather provides a more nuanced picture. The distinction lies in the outcome: our study assessed screening-level depressive symptom burden over the prior 2 weeks (PHQ-9), whereas prior studies focused on a categorical, clinically diagnosed disorder (e.g., MDD) established by structured psychiatric assessment. It is plausible that while the withdrawal-intensifying effect of a high NMR is sufficient to elevate symptom scores on a continuous or semiquantitative scale like the PHQ-9, it may not be a primary etiological driver for the complex, multifactorial syndrome of MDD.^[24]^

Furthermore, our results complement findings from a recent MR study that suggested the causal link from smoking to depression is largely independent of circulating nicotine exposure.^[25,26]^ Our observational, cross-sectional design provided a “snapshot” of the real-time physiological and psychological state of active smokers, where the immediate effects of nicotine clearance and withdrawal are paramount. This represents a different line of evidence from MR studies, which assess the influence of lifelong genetic predispositions.^[27]^ Our study highlighted a specific, behavior-dependent pathway that contributes to the complex interplay between nicotine, smoking, and mood.

### 4.4. Strengths and limitations

This study has several notable strengths. It is based on a large, nationally representative sample, which enhances the generalizability of the findings to the U.S. adult population. The use of a validated, objective biomarker (NMR) for the exposure minimizes potential biases associated with self-reported measures of smoking behavior.^[28]^ The statistical methodology was robust, incorporating complex survey design features and multiple imputation to handle missing data, thereby increasing the validity of the estimates. A key novelty is the formal demonstration of effect modification by smoking status, confirmed through a sensitivity analysis with 2 distinct, biochemically-based definitions of a smoker.

However, the study is also subject to limitations. The cross-sectional design precludes causal inference and leaves temporality unresolved; depressive symptoms could influence smoking behaviors, recent nicotine intake, or cessation attempts (reverse causation), and unmeasured or imperfectly measured factors (e.g., psychiatric treatment, other substance use, stress exposures) may contribute to residual confounding despite comprehensive adjustment. While the proposed biological mechanism (faster metabolism → more severe withdrawal → depressive symptoms) is directional, we cannot rule out reverse causation. The NMR was measured at a single time point, which does not account for potential intraindividual variability over time. The outcome was based on the PHQ-9, a screening instrument for depressive symptoms, not a formal clinical diagnosis of MDD based on a structured psychiatric interview. Finally, despite adjusting for a wide range of potential confounders, residual confounding from unmeasured variables (e.g., use of specific psychotropic medications, other substance use disorders) remains a possibility.

### 4.5. Public health and clinical implications

The findings carry significant implications for both public health and clinical practice. From a clinical perspective, the NMR could serve as a useful biomarker to identify a vulnerable subgroup of smokers – fast metabolizers – who are at a heightened risk for experiencing significant mood disturbances, particularly during attempts to quit. This information could be used to personalize smoking-cessation strategies. For example, fast metabolizers may derive greater benefit from non-nicotine pharmacotherapies, such as varenicline or bupropion, whose efficacy is not dependent on nicotine clearance rates. They may also require more intensive behavioral support focused on managing withdrawal-related negative affect.

From a public health standpoint, these results underscore the importance of integrating mental-health screening and support services into all smoking-cessation programs. Recognizing that a biological trait can predispose certain smokers to more severe mood symptoms during withdrawal highlights the need for proactive, rather than reactive, mental-health care in this population.

## 5. Conclusion

NMR was unrelated to depressive symptoms in the overall population but showed a clear, near-linear positive association among active smokers, consistent across biochemical smoker definitions and sensitivity models. These findings indicate a smoker-specific association and are compatible with an exposure-contingent, hypothesis-generating framework in which faster nicotine clearance may coincide with greater withdrawal-related affect in active smokers, and they suggest that NMR could be explored as a biomarker to inform precision cessation and integrated mental-health support. Prospective longitudinal studies and randomized interventions are needed to establish temporality and directionality, and to determine whether NMR-guided risk stratification and tailored cessation/mental-health support improves clinically meaningful outcomes.

## Author contributions

**Conceptualization:** Ying Zhu, Ruibo Miao.

**Data curation:** Ying Zhu, Ruibo Miao.

**Formal analysis:** Ying Zhu.

**Supervision:** Ruibo Miao.

**Validation:** Ruibo Miao.

**Writing – original draft:** Ying Zhu.

**Writing – review & editing:** Ruibo Miao.

## Supplementary Material




