# Supplementary material for: Nicotine metabolite ratio and clinically significant depressive symptoms in U.S. adults: A smoker-specific association
Source: Medicine (Baltimore). 2026 Apr 10;105(15):e48186. doi: 10.1097/MD.0000000000048186 (PMC13593304; doi:10.1097/MD.0000000000048186)
Supplement: Supplementary file 1 [file medi-105-e48186-s001.pdf]

Supplementary file 1 Table S1. Baseline characteristics by depression status (union; cut=3 ng/mL)

| **Characteristic** | **Overall**<br>N = 2538[1] | **PHQ-9 <10**<br>N = 2187[1] | **PHQ-9 ≥10**<br>N = 351[1] | ***P*** |
|---|---|---|---|---|
| **NMR (3HC/cotinine)** | 0.46 (0.30) | 0.45 (0.30) | 0.50 (0.31) | 0.060 |
| **ln(NMR)** | -0.97 (0.64) | -0.98 (0.64) | -0.88 (0.65) | 0.016 |
| **NMR quartiles (smokers)** | | | | 0.120 |
| Q1 (lowest) | 603 (19%) | 530 (20%) | 73 (16%) | |
| Q2 | 622 (23%) | 541 (24%) | 81 (19%) | |
| Q3 | 641 (28%) | 558 (28%) | 83 (28%) | |
| Q4 (highest) | 672 (30%) | 558 (29%) | 114 (37%) | |
| **Biologic current exposure (LBXCOT ≥ 3 ng/mL)** | | | | 0.100 |
| < 3 ng/mL | 80 (3.7%) | 77 (4.1%) | 3 (1.3%) | |
| LBXCOT ≥ 3 ng/mL | 2,458 (96%) | 2,110 (96%) | 348 (99%) | |
| **PHQ-9 total score** | 4.32 (5.05) | 2.62 (2.67) | 14.40 (4.00) | <0.001 |
| **PHQ-9 severity** | | | | <0.001 |
| None (0–4) | 1,659 (65%) | 1,659 (76%) | 0 (0%) | |
| Mild (5–9) | 528 (21%) | 528 (24%) | 0 (0%) | |
| Moderate (10–14) | 203 (8.8%) | 0 (0%) | 203 (61%) | |
| Mod-severe (15–19) | 99 (3.9%) | 0 (0%) | 99 (27%) | |
| Severe (20–27) | 49 (1.8%) | 0 (0%) | 49 (12%) | |
| **Age (years)** | 43.29 (15.82) | 43.35 (15.90) | 42.90 (15.35) | 0.643 |
| **Sex** | | | | <0.001 |
| Male | 1,550 (60%) | 1,390 (63%) | 160 (41%) | |
| Female | 988 (40%) | 797 (37%) | 191 (59%) | |
| **Race/Ethnicity** | | | | 0.151 |
| Mexican American | 270 (6.8%) | 240 (7.0%) | 30 (5.5%) | |
| Other Hispanic | 186 (4.7%) | 156 (4.5%) | 30 (5.3%) | |

| Characteristic | Overall<br>N = 2538[1] | PHQ-9 <10<br>N = 2187[1] | PHQ-9 ≥10<br>N = 351[1] | *P* |
|---|---|---|---|---|
| Non-Hispanic White | 990 (64%) | 830 (64%) | 160 (64%) | |
| Non-Hispanic Black | 786 (16%) | 697 (16%) | 89 (14%) | |
| Other/Multiracial | 306 (8.7%) | 264 (8.2%) | 42 (12%) | |
| **Education** | | | | 0.002 |
| <9th grade | 167 (4.0%) | 133 (3.5%) | 34 (7.0%) | |
| 9–11th grade | 419 (13%) | 342 (12%) | 77 (18%) | |
| High school/GED | 741 (34%) | 650 (35%) | 91 (30%) | |
| Some college/AA | 827 (34%) | 714 (33%) | 113 (36%) | |
| College graduate+ | 279 (15%) | 256 (16%) | 23 (9.1%) | |
| **PIR** | 2.44 (1.59) | 2.52 (1.60) | 1.90 (1.40) | <0.001 |
| **Marital status** | | | | <0.001 |
| Married | 862 (40%) | 774 (42%) | 88 (28%) | |
| Living with partner | 353 (15%) | 311 (15%) | 42 (11%) | |
| Never married | 613 (24%) | 523 (24%) | 90 (27%) | |
| Widowed | 138 (4.2%) | 115 (3.8%) | 23 (6.5%) | |
| Divorced | 344 (13%) | 273 (12%) | 71 (20%) | |
| Separated | 122 (4.0%) | 98 (3.5%) | 24 (6.8%) | |
| **Any alcohol use in past 12 months** | 2,053 (85%) | 1,771 (85%) | 282 (83%) | 0.392 |
| **Smoking status** | | | | <0.001 |
| Never | 407 (14%) | 381 (16%) | 26 (6.8%) | |
| Former | 374 (18%) | 334 (19%) | 40 (11%) | |
| Current | 1,757 (68%) | 1,472 (66%) | 285 (82%) | |
| **BMI (kg/m²)** | 29.17 (7.31) | 29.06 (7.17) | 29.81 (8.05) | 0.172 |
| **Waist Circumference (cm)** | 99.98 (17.54) | 99.88 (17.35) | 100.59 (18.66) | 0.612 |
| **Sleep duration (hours/night)** | 7.53 (1.67) | 7.50 (1.60) | 7.72 (1.99) | 0.117 |

| Characteristic | Overall<br>N = 2538[1] | PHQ-9 <10<br>N = 2187[1] | PHQ-9 ≥10<br>N = 351[1] | ***P*** |
|---|---|---|---|---|
| **Hypertension (ever told)** | 869 (32%) | 726 (30%) | 143 (41%) | 0.004 |
| **Diabetes (doctor told)** | | | | 0.193 |
| No | 2,190 (90%) | 1,894 (90%) | 296 (88%) | |
| Yes | 287 (8.4%) | 238 (8.1%) | 49 (10%) | |
| Borderline | 60 (1.8%) | 54 (2.0%) | 6 (1.2%) | |
| **History of stroke** | 125 (3.6%) | 93 (2.9%) | 32 (8.1%) | <0.001 |
| **History of CHD** | 97 (3.6%) | 77 (3.3%) | 20 (5.7%) | 0.110 |

[1]Mean (SD); n (unweighted) (%)

NMR = nicotine metabolite ratio; PHQ-9 = Patient Health Questionnaire-9; 3HC = 3-hydroxycotinine; PIR = Poverty-income ratio; BMI = body mass index; CHD = coronary heart disease

Supplementary file 2 Table S2 Baseline characteristics by NMR quartiles (union; cut=10 ng/mL)

| **Characteristic** | **Overall**<br>N = 4181[1] | **Q1 (lowest)**<br>N = 1046[1] | **Q2**<br>N = 1045[1] | **Q3**<br>N = 1045[1] | **Q4 (highest)**<br>N = 1045[1] | ***P*** |
|---|---|---|---|---|---|---|
| **NMR (3HC/cotinine)** | 0.45 (0.29) | 0.17 (0.05) | 0.30 (0.04) | 0.44 (0.04) | 0.79 (0.31) | <0.001 |
| **ln(NMR)** | -0.98 (0.64) | -1.88 (0.48) | -1.20 (0.12) | -0.83 (0.10) | -0.29 (0.30) | <0.001 |
| **Biologic current exposure (LBXCOT ≥ 10 ng/mL)** | | | | | | <0.001 |
| < 10 ng/mL | 172 (4.6%) | 20 (2.7%) | 30 (3.4%) | 30 (2.2%) | 92 (9.3%) | |
| LBXCOT ≥ 10 ng/mL | 4,009 (95%) | 1,026 (97%) | 1,015 (97%) | 1,015 (98%) | 953 (91%) | |
| **PHQ-9 total score** | 4.39 (5.10) | 4.46 (5.19) | 4.14 (4.84) | 4.29 (4.89) | 4.67 (5.42) | 0.535 |
| **PHQ-9 severity** | | | | | | 0.208 |
| None (0–4) | 1,558 (65%) | 383 (65%) | 396 (65%) | 390 (65%) | 389 (63%) | |
| Mild (5–9) | 499 (21%) | 127 (22%) | 129 (22%) | 124 (21%) | 119 (18%) | |
| Moderate (10–14) | 196 (9.0%) | 39 (6.3%) | 45 (6.8%) | 52 (10%) | 60 (11%) | |
| Mod-severe (15–19) | 97 (4.1%) | 24 (3.9%) | 25 (4.7%) | 15 (2.2%) | 33 (5.5%) | |
| Severe (20–27) | 48 (1.8%) | 14 (2.6%) | 9 (1.0%) | 13 (1.9%) | 12 (1.7%) | |
| **Age (years)** | 42.48 (15.92) | 39.26 (15.59) | 40.71 (15.56) | 42.21 (15.50) | 46.52 (16.03) | <0.001 |
| **Sex** | | | | | | <0.001 |
| Male | 2,525 (59%) | 710 (67%) | 666 (64%) | 635 (61%) | 514 (47%) | |
| Female | 1,656 (41%) | 336 (33%) | 379 (36%) | 410 (39%) | 531 (53%) | |
| **Race/Ethnicity** | | | | | | <0.001 |
| Mexican American | 410 (6.5%) | 91 (7.5%) | 104 (7.1%) | 113 (6.7%) | 102 (5.0%) | |
| Other Hispanic | 283 (4.5%) | 63 (5.2%) | 73 (5.1%) | 74 (4.3%) | 73 (3.6%) | |
| Non-Hispanic White | 1,798 (66%) | 292 (49%) | 424 (62%) | 486 (69%) | 596 (77%) | |
| Non-Hispanic Black | 1,204 (15%) | 409 (26%) | 356 (19%) | 256 (12%) | 183 (7.6%) | |
| Other/Multiracial | 486 (8.3%) | 191 (12%) | 88 (6.4%) | 116 (8.8%) | 91 (6.4%) | |
| **Education** | | | | | | 0.154 |
| <9th grade | 269 (4.0%) | 59 (4.7%) | 67 (4.1%) | 65 (3.2%) | 78 (4.2%) | |
| 9–11th grade | 732 (15%) | 195 (18%) | 179 (16%) | 191 (14%) | 167 (12%) | |
| High school/GED | 1,175 (33%) | 286 (31%) | 288 (33%) | 301 (37%) | 300 (31%) | |
| Some college/AA | 1,294 (34%) | 300 (33%) | 323 (34%) | 332 (34%) | 339 (37%) | |
| College graduate+ | 427 (14%) | 109 (14%) | 95 (13%) | 96 (13%) | 127 (16%) | |
| **PIR** | 2.37 (1.59) | 2.18 (1.51) | 2.35 (1.61) | 2.42 (1.58) | 2.49 (1.63) | 0.011 |
| **Marital status** | | | | | | 0.011 |
| Married | 1,446 (40%) | 340 (40%) | 334 (39%) | 376 (40%) | 396 (43%) | |
| Living with partner | 523 (14%) | 130 (13%) | 132 (15%) | 141 (13%) | 120 (13%) | |
| Never married | 977 (25%) | 306 (31%) | 269 (27%) | 214 (23%) | 188 (20%) | |
| Widowed | 207 (4.2%) | 27 (2.1%) | 33 (2.8%) | 60 (5.2%) | 87 (5.8%) | |
| Divorced | 562 (13%) | 111 (10%) | 130 (13%) | 150 (15%) | 171 (14%) | |
| Separated | 184 (3.9%) | 36 (3.4%) | 54 (4.2%) | 45 (3.9%) | 49 (4.1%) | |
| **Any alcohol use in past 12 months** | 3,102 (86%) | 761 (88%) | 770 (86%) | 791 (86%) | 780 (85%) | 0.583 |
| **Smoking status** | | | | | | 0.016 |
| Never | 521 (12%) | 156 (13%) | 159 (14%) | 119 (13%) | 87 (7.4%) | |

| Characteristic | Overall<br>N = 4181[1] | Q1 (lowest)<br>N = 1046[1] | Q2<br>N = 1045[1] | Q3<br>N = 1045[1] | Q4 (highest)<br>N = 1045[1] | *P* |
|---|---|---|---|---|---|---|
| Former | 519 (15%) | 125 (16%) | 114 (12%) | 130 (15%) | 150 (17%) | |
| Current | 3,017 (73%) | 716 (70%) | 738 (74%) | 766 (72%) | 797 (75%) | |
| **BMI (kg/m²)** | 28.83 (7.18) | 29.03 (7.22) | 29.50 (7.60) | 28.85 (7.17) | 28.11 (6.74) | 0.005 |
| **Waist Circumference (cm)** | 99.05 (17.51) | 98.70 (17.36) | 100.54 (18.83) | 99.52 (17.75) | 97.60 (16.07) | 0.096 |
| **Sleep duration (hours/night)** | 7.27 (1.69) | 7.25 (1.71) | 7.18 (1.72) | 7.17 (1.62) | 7.44 (1.69) | 0.007 |
| **Hypertension (ever told)** | 1,406 (32%) | 302 (28%) | 298 (28%) | 344 (30%) | 462 (41%) | <0.001 |
| **Diabetes (doctor told)** | | | | | | 0.001 |
| No | 3,642 (90%) | 937 (91%) | 945 (92%) | 898 (91%) | 862 (86%) | |
| Yes | 446 (8.6%) | 89 (7.2%) | 83 (6.8%) | 120 (7.1%) | 154 (12%) | |
| Borderline | 91 (1.8%) | 20 (2.0%) | 16 (0.9%) | 27 (2.2%) | 28 (2.1%) | |
| **History of stroke** | 179 (3.4%) | 51 (4.0%) | 23 (2.0%) | 38 (2.6%) | 67 (4.6%) | 0.039 |
| **History of CHD** | 154 (3.9%) | 26 (2.7%) | 26 (2.8%) | 36 (3.8%) | 66 (5.7%) | 0.027 |

[1]Mean (SD); n (unweighted) (%)

NMR = nicotine metabolite ratio; PHQ-9 = Patient Health Questionnaire-9; 3HC = 3-hydroxycotinine; PIR = Poverty-income ratio; BMI = body mass index; CHD = coronary heart disease

Supplementary file 3 Table S3 Baseline characteristics by NMR quartiles (Overall Adults)

| Characteristic | Overall<br>N = 22796[1] | Q1 (lowest)<br>N = 5699[1] | Q2<br>N = 5699[1] | Q3<br>N = 10808[1] | Q4 (highest)<br>N = 590[1] | *P* |
|---|---|---|---|---|---|---|
| **NMR (3HC/cotinine)** | 0.68 (0.39) | 0.23 (0.07) | 0.44 (0.07) | 0.91 (0.15) | 1.51 (0.95) | <0.001 |
| **ln(NMR)** | -0.57 (0.64) | -1.55 (0.43) | -0.83 (0.15) | -0.11 (0.19) | 0.34 (0.32) | <0.001 |
| **Biologic current exposure (LBXCOT ≥ 3 ng/mL)** | | | | | | <0.001 |
| Low/none | 18,266 (78%) | 3,655 (58%) | 4,097 (66%) | 10,071 (92%) | 443 (72%) | |
| Active exposure (LBXCOT ≥ cut) | 4,530 (22%) | 2,044 (42%) | 1,602 (34%) | 737 (8.0%) | 147 (28%) | |
| **PHQ-9 total score** | 3.13 (4.06) | 3.71 (4.56) | 3.42 (4.26) | 2.74 (3.73) | 3.88 (4.06) | <0.001 |
| **PHQ-9 severity** | | | | | | <0.001 |
| None (0–4) | 7,198 (76%) | 1,542 (71%) | 1,748 (72%) | 3,698 (80%) | 210 (64%) | |
| Mild (5–9) | 1,650 (16%) | 402 (19%) | 444 (18%) | 736 (14%) | 68 (25%) | |
| Moderate (10–14) | 522 (5.2%) | 129 (5.4%) | 145 (6.4%) | 222 (4.4%) | 26 (8.9%) | |
| Mod-severe (15–19) | 217 (2.0%) | 65 (3.2%) | 51 (2.0%) | 92 (1.6%) | 9 (2.3%) | |
| Severe (20–27) | 94 (0.8%) | 29 (1.3%) | 30 (1.0%) | 33 (0.5%) | 2 (0.2%) | |
| **Age (years)** | 40.66 (21.30) | 33.81 (18.86) | 38.13 (20.08) | 43.61 (21.85) | 55.20 (19.26) | <0.001 |
| **Sex** | | | | | | <0.001 |
| Male | 11,132 (49%) | 3,191 (58%) | 2,915 (53%) | 4,805 (44%) | 221 (37%) | |
| Female | 11,664 (51%) | 2,508 (42%) | 2,784 (47%) | 6,003 (56%) | 369 (63%) | |
| **Race/Ethnicity** | | | | | | <0.001 |
| Mexican American | 3,960 (11%) | 691 (9.8%) | 859 (9.8%) | 2,352 (12%) | 58 (4.2%) | |
| Other Hispanic | 2,421 (6.7%) | 441 (6.4%) | 583 (6.6%) | 1,361 (7.2%) | 36 (2.6%) | |
| Non-Hispanic White | 7,764 (62%) | 1,407 (48%) | 1,982 (60%) | 4,007 (67%) | 368 (82%) | |
| Non-Hispanic Black | 4,925 (11%) | 2,134 (24%) | 1,330 (13%) | 1,378 (6.2%) | 83 (5.9%) | |
| Other/Multiracial | 3,726 (9.5%) | 1,026 (12%) | 945 (11%) | 1,710 (8.1%) | 45 (5.5%) | |
| **Education** | | | | | | <0.001 |
| <9th grade | 1,457 (4.8%) | 274 (5.1%) | 340 (4.8%) | 793 (4.7%) | 50 (4.3%) | |

| Characteristic | Overall<br>N = 22796[1] | Q1 (lowest)<br>N = 5699[1] | Q2<br>N = 5699[1] | Q3<br>N = 10808[1] | Q4 (highest)<br>N = 590[1] | ***P*** |
|---|---|---|---|---|---|---|
| 9–11th grade | 1,891 (8.8%) | 539 (13%) | 548 (11%) | 724 (6.4%) | 80 (10%) | |
| High school/GED | 3,498 (23%) | 921 (28%) | 969 (28%) | 1,442 (19%) | 166 (32%) | |
| Some college/AA | 4,795 (32%) | 1,156 (36%) | 1,231 (34%) | 2,248 (30%) | 160 (30%) | |
| College graduate+ | 3,809 (31%) | 593 (18%) | 756 (22%) | 2,355 (40%) | 105 (24%) | |
| **PIR** | 2.88 (1.66) | 2.31 (1.59) | 2.60 (1.63) | 3.24 (1.62) | 2.75 (1.57) | <0.001 |
| **Marital status** | | | | | | <0.001 |
| Married | 7,920 (55%) | 1,418 (42%) | 1,782 (48%) | 4,478 (63%) | 242 (47%) | |
| Living with partner | 1,320 (8.5%) | 404 (13%) | 406 (11%) | 476 (6.2%) | 34 (7.5%) | |
| Never married | 2,813 (18%) | 995 (29%) | 792 (22%) | 956 (13%) | 70 (14%) | |
| Widowed | 1,163 (5.8%) | 141 (2.9%) | 238 (4.7%) | 681 (6.7%) | 103 (16%) | |
| Divorced | 1,723 (10%) | 392 (10%) | 481 (12%) | 764 (9.4%) | 86 (13%) | |
| Separated | 520 (2.5%) | 135 (3.2%) | 152 (3.2%) | 207 (1.8%) | 26 (2.8%) | |
| **Any alcohol use in past 12 months** | 10,292 (78%) | 2,499 (81%) | 2,676 (80%) | 4,758 (75%) | 359 (77%) | <0.001 |
| **Smoking status** | | | | | | <0.001 |
| Never | 9,555 (58%) | 1,813 (45%) | 2,084 (49%) | 5,392 (67%) | 266 (45%) | |
| Former | 3,661 (24%) | 677 (19%) | 851 (23%) | 1,942 (26%) | 191 (35%) | |
| Current | 3,017 (18%) | 1,251 (36%) | 1,099 (28%) | 556 (7.2%) | 111 (20%) | |
| **BMI (kg/m²)** | 27.83 (7.64) | 28.09 (8.24) | 27.82 (7.78) | 27.68 (7.35) | 28.74 (7.18) | 0.040 |
| **Waist Circumference (cm)** | 94.77 (20.21) | 94.45 (21.68) | 94.57 (20.42) | 94.72 (19.63) | 99.12 (17.36) | <0.001 |
| **Sleep duration (hours/night)** | 7.44 (1.47) | 7.29 (1.62) | 7.34 (1.53) | 7.52 (1.37) | 7.76 (1.65) | <0.001 |
| **Hypertension (ever told)** | 5,846 (31%) | 1,118 (27%) | 1,366 (29%) | 3,023 (33%) | 339 (52%) | <0.001 |
| **Diabetes (doctor told)** | | | | | | <0.001 |
| No | 20,034 (89%) | 5,231 (92%) | 5,064 (91%) | 9,328 (88%) | 411 (76%) | |
| Yes | 2,279 (8.9%) | 384 (6.6%) | 513 (7.3%) | 1,222 (9.8%) | 160 (20%) | |
| Borderline | 470 (2.1%) | 83 (1.5%) | 116 (1.8%) | 253 (2.4%) | 18 (3.5%) | |

| Characteristic | Overall<br>N = 22796[1] | Q1 (lowest)<br>N = 5699[1] | Q2<br>N = 5699[1] | Q3<br>N = 10808[1] | Q4 (highest)<br>N = 590[1] | ***P*** |
|---|---|---|---|---|---|---|
| **History of stroke** | 614 (3.0%) | 122 (2.5%) | 136 (2.7%) | 303 (2.9%) | 53 (7.3%) | <0.001 |
| **History of CHD** | 659 (3.8%) | 84 (2.1%) | 126 (3.1%) | 390 (4.2%) | 59 (9.8%) | <0.001 |

[1]Mean (SD); n (unweighted) (%)

NMR = nicotine metabolite ratio; PHQ-9 = Patient Health Questionnaire-9; 3HC = 3-hydroxycotinine; PIR = Poverty-income ratio; BMI = body mass index; CHD = coronary heart disease
