# Supplementary material for: Nicotine metabolite ratio and clinically significant depressive symptoms in U.S. adults: A smoker-specific association
Source: Medicine (Baltimore). 2026 Apr 10;105(15):e48186. doi: 10.1097/MD.0000000000048186 (PMC13593304; doi:10.1097/MD.0000000000048186)
Supplement: Supplementary file 2 [file medi-105-e48186-s002.pdf]

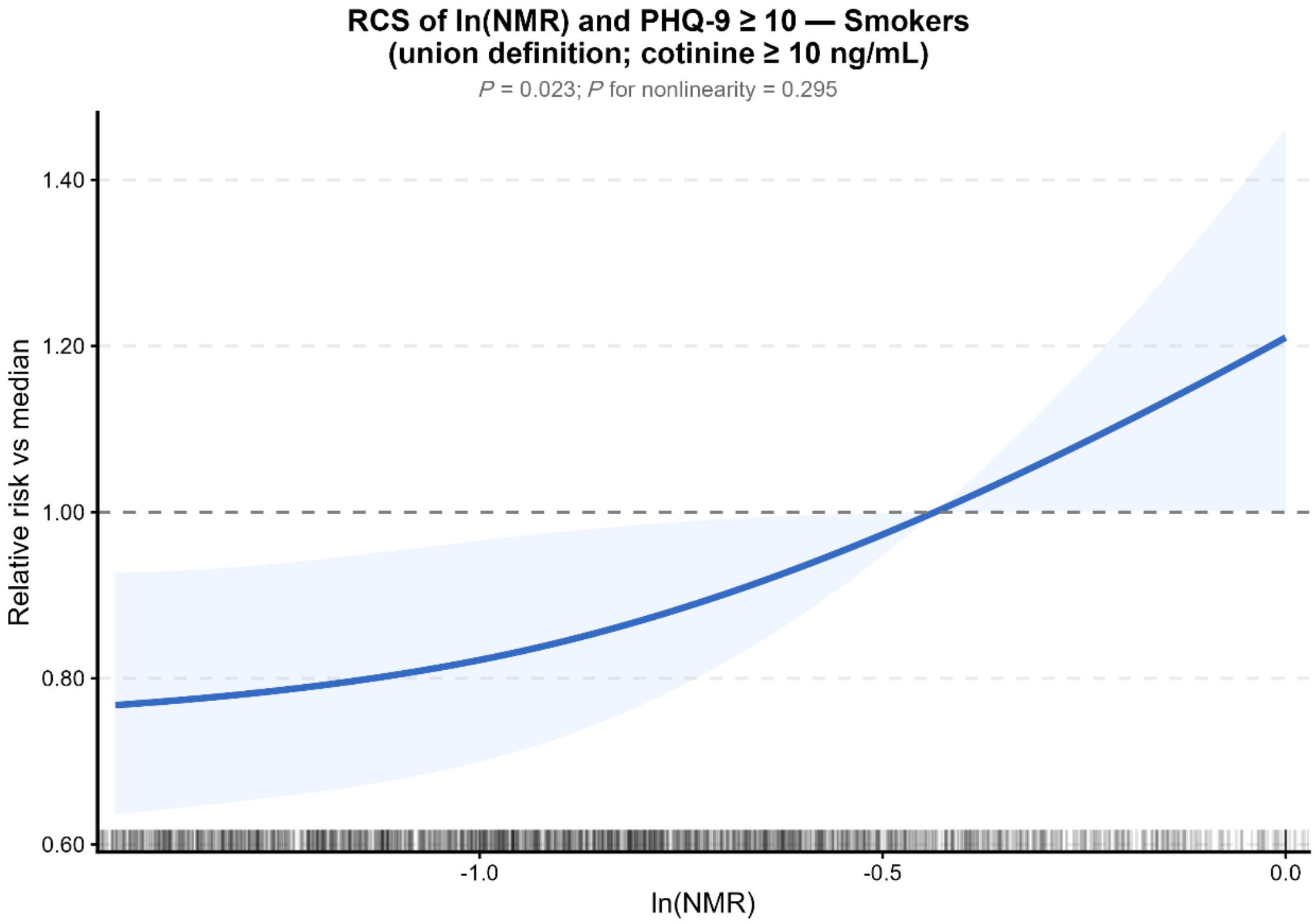

Supplementary file 4 Figure S1. Restricted cubic spline of ln(NMR) versus PHQ-9≥10 among smokers (union; cotinine ≥10 ng/mL); risk shown relative to the median.

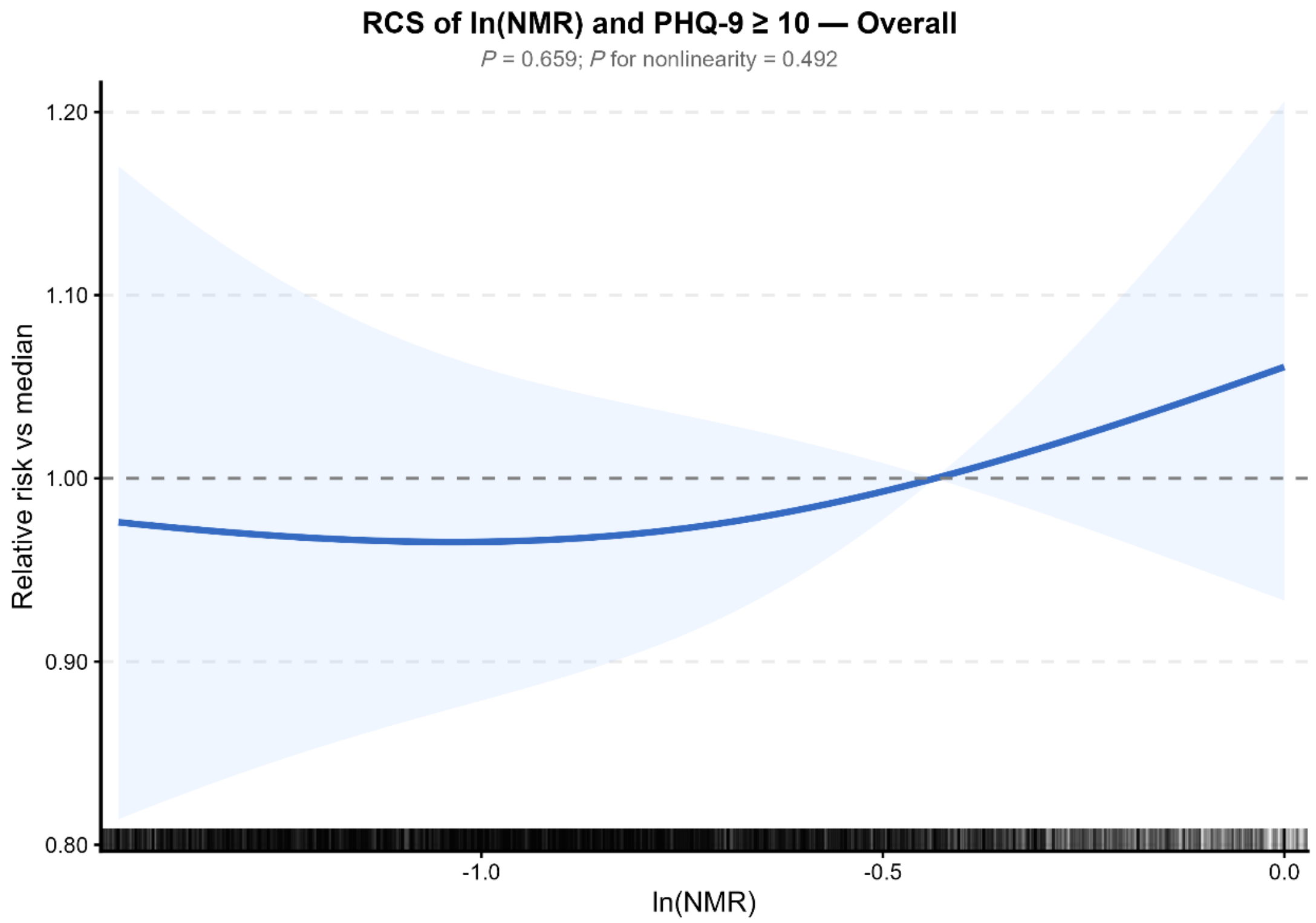

Supplementary file 5 Figure S2. Restricted cubic spline of ln(NMR) versus PHQ-9≥10 in the overall adult population; risk shown relative to the median.

Association per 1-SD increase in ln(NMR) with clinically significant depressive symptoms (PHQ-9 ≥ 10): prespecified subgroup analyses among smokers (union definition; cotinine ≥ 10 ng/mL)

	RR (95% CI)	*P* value	*P* for interaction	*P* for interaction (BH-FDR)
Age			0.003	0.004
<50 years	1.139 (0.943, 1.376)	0.177		
≥50 years	1.244 (1.057, 1.464)	0.009		
Alcohol use			0.447	0.459
No	1.213 (0.989, 1.489)	0.064		
Yes	1.185 (1.015, 1.383)	0.032		
BMI			<0.001	<0.001
25–29.9	1.020 (0.813, 1.280)	0.865		
<25	1.485 (1.189, 1.854)	<0.001		
≥30	1.103 (0.886, 1.371)	0.381		
CHD			0.459	0.459
No	1.188 (1.047, 1.349)	0.008		
Yes	1.252 (0.693, 2.262)	0.456		
Cotinine (tertiles)			<0.001	<0.001
T1 (low)	1.269 (1.015, 1.585)	0.036		
T2	0.974 (0.828, 1.146)	0.751		
T3 (high)	1.400 (1.071, 1.832)	0.014		
Diabetes			<0.001	<0.001
No	1.228 (1.082, 1.394)	0.001		
Yes	0.963 (0.702, 1.322)	0.817		
Borderline	1.189 (0.412, 3.430)	0.749		
Hypertension			0.010	0.013
No	1.143 (0.961, 1.359)	0.131		
Yes	1.265 (0.986, 1.624)	0.065		
PIR (tertiles)			<0.001	<0.001
T1 (low)	0.917 (0.796, 1.056)	0.231		
T2	1.444 (1.159, 1.799)	0.001		
T3 (high)	1.463 (1.151, 1.859)	0.002		
Race/Ethnicity			<0.001	<0.001
Mexican American	0.789 (0.632, 0.986)	0.037		
Non-Hispanic Black	1.129 (0.953, 1.337)	0.159		
Non-Hispanic White	1.211 (1.004, 1.461)	0.045		
Other Hispanic	0.744 (0.525, 1.053)	0.095		
Sex			0.001	0.002
Female	1.069 (0.909, 1.256)	0.420		
Male	1.217 (0.935, 1.584)	0.145		
Stroke			<0.001	<0.001
No	1.220 (1.081, 1.377)	0.001		
Yes	0.960 (0.772, 1.193)	0.712		

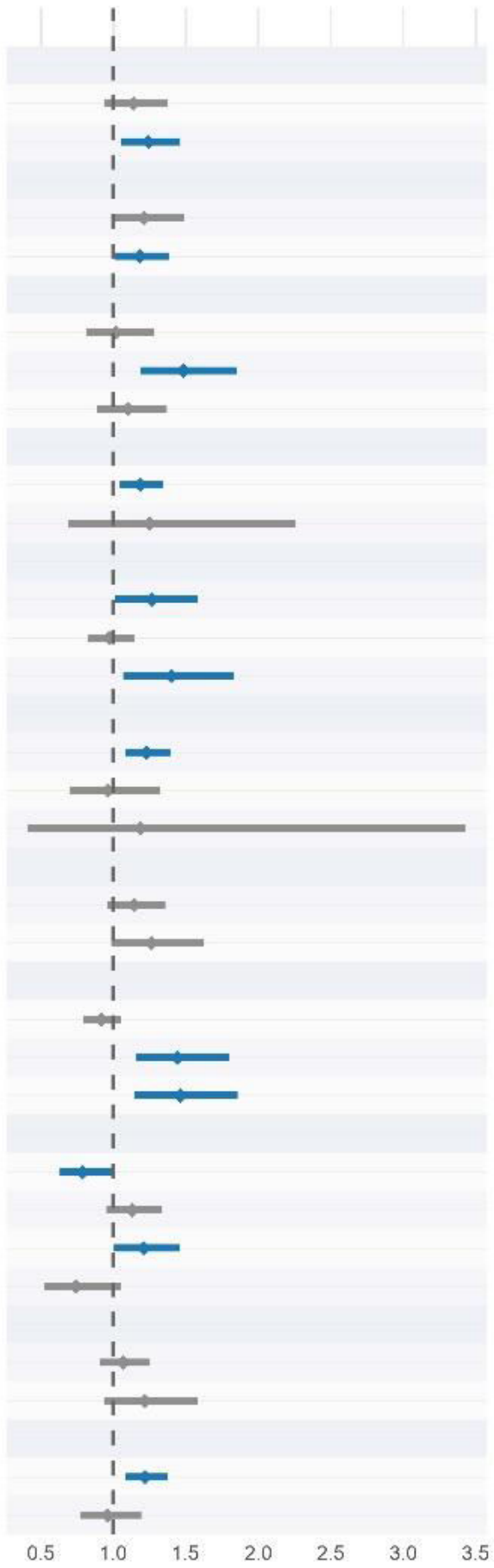

Supplementary file 6 Figure S3. Prespecified subgroup analyses among smokers using a stricter definition (serum cotinine ≥10 ng/mL): forest plot of per-SD ln(NMR) relative risks with *P* for interaction.

Association per 1-SD increase in ln(NMR) with clinically significant depressive symptoms (PHQ-9 ≥ 10): prespecified subgroup analyses in the overall population

	RR (95% CI)	*P* value	*P* for interaction	*P* for interaction (BH-FDR)
Age			<0.001	<0.001
<50 years	0.815 (0.737, 0.902)	<0.001		
≥50 years	0.944 (0.835, 1.068)	0.358		
Alcohol use			<0.001	<0.001
No	0.985 (0.854, 1.135)	0.830		
Yes	0.835 (0.755, 0.923)	<0.001		
BMI			<0.001	<0.001
25–29.9	0.806 (0.729, 0.891)	<0.001		
<25	0.911 (0.774, 1.074)	0.268		
≥30	0.862 (0.760, 0.977)	0.020		
CHD			0.011	0.013
No	0.860 (0.785, 0.942)	0.001		
Yes	0.955 (0.674, 1.354)	0.796		
Cotinine (tertiles)			<0.001	<0.001
T1 (low)	0.912 (0.810, 1.026)	0.127		
T2	0.933 (0.794, 1.097)	0.403		
T3 (high)	1.386 (1.061, 1.812)	0.017		
Diabetes			<0.001	<0.001
No	0.855 (0.781, 0.936)	<0.001		
Yes	0.867 (0.691, 1.087)	0.216		
Borderline	1.457 (0.847, 2.507)	0.174		
Hypertension			<0.001	<0.001
No	0.805 (0.723, 0.896)	<0.001		
Yes	0.973 (0.872, 1.086)	0.623		
PIR (tertiles)			<0.001	<0.001
T1 (low)	0.911 (0.819, 1.013)	0.086		
T2	0.985 (0.850, 1.143)	0.846		
T3 (high)	0.880 (0.699, 1.107)	0.275		
Race/Ethnicity			<0.001	<0.001
Mexican American	0.811 (0.655, 1.005)	0.056		
Non-Hispanic Black	0.889 (0.787, 1.005)	0.059		
Non-Hispanic White	0.852 (0.747, 0.971)	0.016		
Other Hispanic	0.722 (0.571, 0.915)	0.007		
Sex			0.293	0.330
Female	0.828 (0.755, 0.908)	<0.001		
Male	0.847 (0.714, 1.005)	0.057		
Smoking			<0.001	<0.001
Current	1.158 (1.022, 1.312)	0.021		
Former	1.027 (0.827, 1.275)	0.811		
Never	1.068 (0.860, 1.326)	0.552		
Stroke			<0.001	<0.001
No	0.873 (0.796, 0.957)	0.004		
Yes	0.766 (0.663, 0.885)	<0.001		

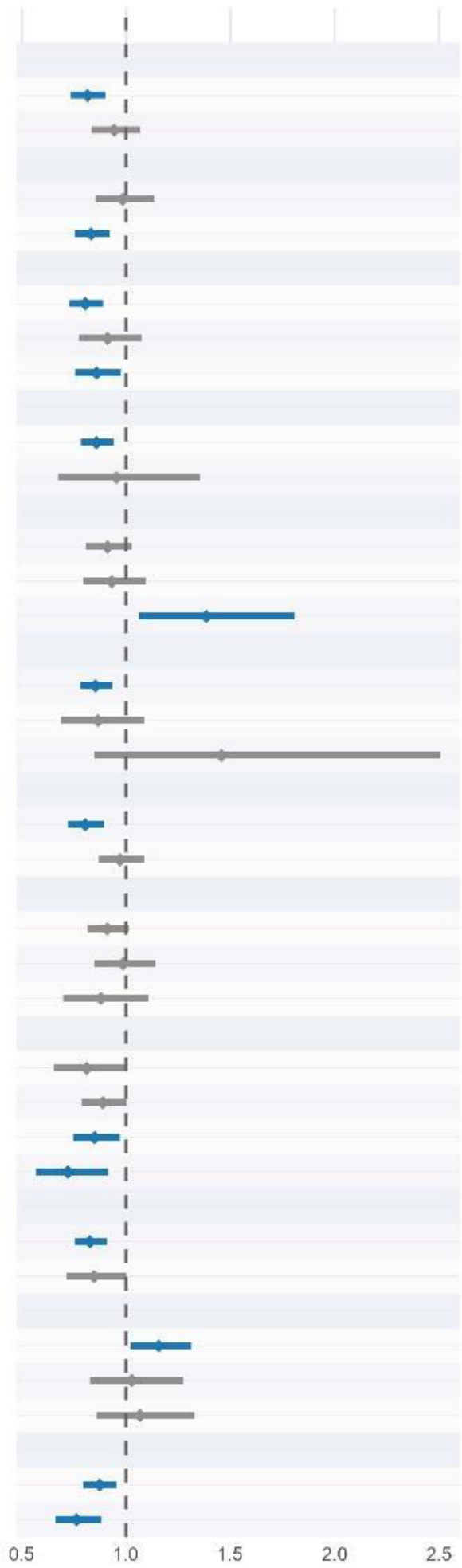

Supplementary file 7 Figure S4. Prespecified subgroup analyses in the overall adult population: forest plot of per-SD ln(NMR) relative risks with *P* for interaction.
